# Gender-and age-specific associations of sleep duration and quality with cognitive impairment in community-dwelling older adults in Anhui Province, China

**DOI:** 10.3389/fpubh.2023.1047025

**Published:** 2024-01-05

**Authors:** Xuechun Liu, Peiru Xu, Rong Wei, Beijing Cheng, Liang Sun, Linsheng Yang, Guihai Chen

**Affiliations:** ^1^Department of Neurology, The Second People’s Hospital of Hefei, Hefei, China; ^2^Anhui Provincial Center for Disease Control and Prevention, Hefei, China; ^3^Outpatient Department of the Second Hospital of Anhui Medical University, Hefei, China; ^4^School of Public Health, Anhui Medical University, Hefei, China; ^5^Fuyang Center of Disease Control and Prevention, Fuyang, China; ^6^Department of Neurology (Sleep Disorders), The Affiliated Chaohu Hospital of Anhui Medical University, Hefei, China

**Keywords:** sleep duration, sleep quality, cognitive test performance, cognitive impairment, older adult

## Abstract

**Objective:**

To examine associations of sleep duration and quality with cognitive impairment in older adults and the moderating role of gender and age in these associations.

**Methods:**

This community-based cross-sectional study included 4,837 participants aged 60 years and above. Cognitive function was assessed using the Chinese version of the Mini-Mental State Examination (MMSE), and the participants were grouped based on the presence of cognitive impairment. The duration and quality of sleep were assessed using the Pittsburgh Sleep Quality Index (PSQI). Multivariate logistic regression models were used to analyze associations of sleep duration and quality with cognitive impairment. The role of age and gender in these associations have also been explored.

**Results:**

The age (mean ± SD) of the participants was 71.13 ± 5.50 years. Of all older adults, 1,811 (37.44%) were detected as cognitive impairment, and 1755 (36.8%) had poor sleep quality. Among those with cognitive impairment, 51.09% were female. The proportion of the participants with cognitive impairment is significantly higher in those with symptoms of depression (49.73%, 273/549) (*χ*^2^ = 41.275, *p* < 0.001) than in those without depressive symptoms. After adjustment for multiple confounding factors and the crucial covariate (depressive symptoms), the odds ratios (OR) (95% confidence interval [CI]) of cognitive impairment (with 7–7.9 h regarded as the reference group) for individuals with a sleep duration of <6, 6–6.9, 8–8.9, and ≥ 9 h were 1.280 (1.053–1.557), 1.425 (1.175–1.728), 1.294 (1.068–1.566), and 1.360 (1.109–1.668), respectively. Subgroup analysis showed a V-shaped association between night sleep duration and cognitive impairment in males (*p* ≤ 0.05), and the association was stronger for individuals aged 60–80 years. With regard to sleep quality, the fully adjusted OR (95%CI) of cognitive impairment were 1.263 (1.108–1.440). According to scores of subscales in the PSQI, daytime dysfunction was associated with an increased risk of cognitive impairment (OR: 1.128, 95%CI: 1.055–1.207). Subgroup analysis also revealed a statistically significant correlation between poor sleep quality (including daytime dysfunction) and cognitive impairment in different gender and age groups, with the association being stronger in females (OR: 1.287, 95%CI: 1.080–1.534) and those aged 81–97 years (OR: 2.128, 95%CI: 1.152–3.934). For cognitive impairment, the group aged 81–97 years with daytime dysfunction was associated with a higher odds ratio than other age groups.

**Conclusion:**

The present study showed that inadequate or excessive sleep was associated with cognitive impairment, especially in males, who exhibited a V-shaped association. Cognitive impairment was also associated with poor sleep quality as well as daytime dysfunction, with females and individuals aged 81–97 years exhibiting the strongest association.

## 1 Introduction

Diseases related to cognitive impairment in older adults, such as dementia [including Alzheimer’s disease (AD), vascular dementia and other types of dementia] and mild cognitive impairment (MCI), have become a serious public health concern. It was estimated that 50 million people lived with dementia across the world (1). According to a recent study, the prevalence of dementia among individuals aged 65 years or above was approximately 5.60% in China (2). It is believed that MCI is a transitional stage between intact cognition and dementia. A recent study estimated that MCI could be found in 15.5% of people aged ≥60 years, accounting for 38.77 million people in China (3). Dementia and MCI share similar risk factors, including advanced age, gender, multimorbidity and sleep-related variables and so on. Sleep *per se* is a modifiable lifestyle factor and good sleep can be a protective factor against cognitive impairment. During the long-term pre-clinical stage of dementia, accelerated cognitive impairment is regarded as a cardinal marker (4). Older age is associated with complex changes in sleep patterns and an increased risk of cognitive impairment (5). The older adult population are not only at a higher risk of developing cognitive disorders, but are also particularly vulnerable to sleeping problems (6), which can affect their cognitive processes (7).

Sleep disorders have been a public health problem in both developing and developed countries (8). Both the duration and quality of sleep are important influencing factors for the cognitive function of older adults. Previous studies revealed that sleep duration, including inadequate sleep (9) and excessive sleep (10–12), was associated with an increased risk of cognitive impairment (13). For middle-aged and older individuals, the potential modifiable risk factors of cognitive impairment and dementia include insomnia and the duration of nighttime sleep (14). Previous longitudinal studies revealed a significant V-shaped (15) or U-shaped (16–18) association between sleep duration and cognitive impairment among adults. A few studies have also shown that poor sleep quality was associated with a higher risk of cognitive impairment (19, 20). However, the association between sleep quality and cognitive performance is inconsistent across different studies (21, 22). Significant differences have been found in the duration and quality of sleep between countries with different levels of economic development (23). Notably, most of the studies on the relationship between sleep and dementia were conducted among populations in North America and Europe (24–26). More studies with representative samples are still needed to investigate the contribution of the duration and quality of sleep to cognitive function in older adults in China given that China are experiencing rapid population aging.

It has been found that extreme nighttime sleep duration and poor sleep quality are independently and interactively related to aggravated depressive symptoms (27), and the duration and quality of nocturnal sleep are independent of but also interactive with depression, and cognitive disorders of older people can be modulated by their emotions (28). However, most of the prior studies focused on the influence of either sleep duration or sleep quality and were often lack of adjustment for depressive symptoms. Furthermore, the effects of sleep and depression on cognitive function in older adults were modified by gender and age (29, 30); thus, it is still unclear to what extent these two factors independently affect the cognitive performance in people of different genders and ages. For this reason, we conducted a large-scale, community-based study on older adults to investigate the relationship of both nighttime sleep duration and sleep quality on cognitive function, as well as the independent association stratified by gender and age.

## 2 Methods

### 2.1 Subjects

All the data were collected from the baseline survey of a cohort of older adults in a study on controllable environmental factors and healthy aging, which was launched in Fuyang city, Anhui province, China, from June to August 2018. This cohort was established by the School of Public Health of Anhui Medical University and the local Center for Disease Control and Prevention. Firstly, the multistage stratified random and probability proportionate to size (PPS) sampling method was used to select participants, who were from all the 3 districts and 5 counties in Fuyang city. Secondly, the sampling frame included 3 levels, i.e., district/county, street/town, and community/village. The number of households selected at each primary sampling site (community/village) was proportional to the population size in the street/town and district/county. Four administrative villages were selected from each town, and four towns were selected from each county, with one in the east, west, north and south of the town/county, respectively. Finally, the participants from a total of 8 districts/counties, including 9 communities and 33 towns, were interviewed for the survey.

**Figure 1:**
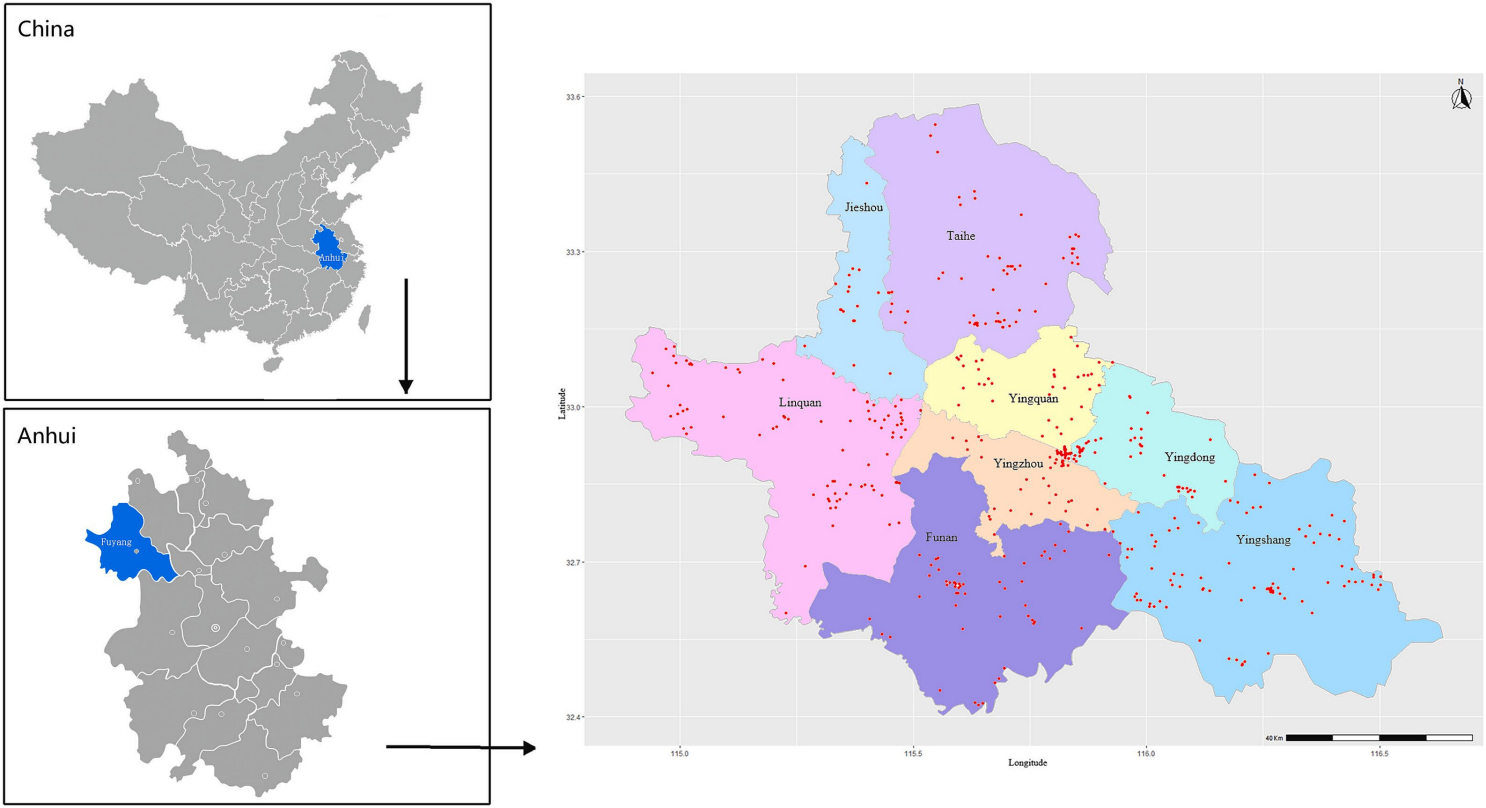
Regional distribution of residence addresses of the study subjects in Fuyang city, Anhui province, China. Red dots represent the residence addresses (*n* = 451) of the study subjects.

The survey was conducted through an extensive in-person one-on-one interview by trained investigators after written inform consent was obtained. All the participants were invited to participate in a free physical examination in the local community hospital. The inclusion criteria were as follows: (1) aged 60 years or older, (2) living in the current place of residence for at least half a year, (3) conscious and able to complete the questionnaire, and (4) no serious neuropsychiatric disease. The exclusion criteria were as follows, (1) having communication barriers, or (2) living with serious neuropsychiatric disease such as dementia. All participants with self-reported dementia were examined by a neurologist based on the criteria provided in Diagnostic and Statistical Manual of Mental Disorders, Fourth Edition (DSM-IV), and those with confirmed dementia were excluded. Individuals with other mental diseases, such as severe psychiatric disorders (e.g., schizophrenia and bipolar disorders), neurological disorders (e.g., Parkinson’s disease and epilepsy), brain tumors, previous history of brain trauma, and severe cognition-affecting diseases (e.g., hepatic failure) were also excluded. Further excluding individuals with incomplete information on sleep parameters (*n* = 59) and cognitive screening (*n* = 243), as well as those under 60 years of age (*n* = 47), 4,837 participants were included in the final analyzes. The detailed distribution of the investigated population of all the districts and counties is presented in Figure 1. This study was approved by the Ethics Committee of Anhui Medical University (No. 20190288).

### 2.2 Cognitive performance

The participants’ cognitive function was examined by trained investigators using the Chinese version of the Mini-Mental State Examination (MMSE) (31), which has been widely used in previous studies (13, 21). The total score of MMSE ranges from 0 to 30, with higher scores reflecting better cognitive function. A considerable proportion of the participants were found to be illiterate (54.33%). As previous studies have found that the level of education is a strong demographic factor affecting the MMSE score and that lower education levels could adversely affect the MMSE score, the optimal cut-off points to define cognitive impairment were determined based on the participants’ level of education (31, 32), with a cut-off point of 16/17 for illiterate individuals, 19/20 for those with primary school education, and 23/24 for those with higher education levels.

### 2.3 Assessment of sleep-related variables

The PSQI scale (33) was used to assesses individual’s sleep quality during the past month. The scale consists of seven components: sleep duration, subjective sleep quality, sleep latency, sleep efficiency, sleep disturbance, using of sleeping medications, and daytime dysfunction, with each of them scoring 0–3. Thus, the global PSQI score ranges from 0 to 21, with higher scores indicating poorer quality of sleep (34) and the cut-off point for poor sleep quality being 7 (35).

The baseline nighttime sleep duration was obtained through a face-to-face patient interview. To make this information as reliable as possible, sleep duration was defined as hours of actual sleep at night. According to the PSQI, we asked the following question in the first place: “During the past month, how many hours of actual sleep did you usually have at night?” Based on the reported sleep duration, the following questions, i.e., “When do you usually go to bed at night?” “How long [in minutes] does it usually take you to fall asleep each night?” and “When do you usually get up in the morning?” were then asked. With a standard approach (36), the sleep duration was categorized as <6 h., 6–6.9 h., 7–7.9 h, 8–8.9 h. and ≥ 9 h. The sleep duration of 7–7.9 h. per night was regarded as the reference (37), because it has been identified as the optimal sleep duration in healthy adults (38–43). Generally, 7–8 h. is considered as the optimal sleep duration (44).

### 2.4 Measurement of covariates

Potential confounding covariates were collected using questionnaires, which included demographic factors (gender, age, place of residence, education, occupation, marital status, living arrangements, and economic situation), factors related to health behaviors (consumption of staple food and vegetables, smoking, drinking, and BMI), factors related to chronic diseases (diabetes, hypertension, CHD, stroke, and COPD) and depressive symptoms.

The participants were stratified by age (60–70 years, 71–80 years, and 81–97 years), residence area (rural and urban), and level of education [illiteracy (0 year of education), primary school (1–6 years), and junior school or above (>6 years)]. Other factors included occupation (employed knowledge worker, employed manual worker, and others), marital status [married and single (including widowed and divorced)], economic status (affluent, ordinary, needy), alcohol drinking status (never, occasionally, and every day), and smoking status (non-smoker, former smoker and current smoker). The participants’ height and weight were measured, and their BMI (kg/m^2^) was calculated and stratified (<18.5, 18.5–23.99, 24.0–27.99, and ≥ 28.0).

With regard to chronic diseases, diabetes mellitus (DM) was confirmed when the fasting plasma glucose concentration was ≥7.0 mmol/L, with self-report of a history of diabetes and treatment with insulin or other oral medications. Hypertension was defined based on the use of antihypertensive medication, a systolic blood pressure (SBP) of >140 mmHg, a diastolic blood pressure (DBP) of >90 mmHg (measured by trained physicians), or a self-report history of hypertension. Coronary heart diseases (CHDs) included myocardial infarction, as well as a history of coronary angioplasty or coronary artery bypass surgery. The participants were grouped based on whether they had diabetes, hypertension, CHDs, stroke, and chronic obstructive pulmonary disease (COPD), respectively. Depressive symptoms were assessed using the Chinese version of the 15-item Geriatric Depression Scale (GDS). The GDS score ranges from 0 to 15, with higher scores indicating higher severity of depressive symptoms. The participants were also grouped based on whether they had depressive symptoms, with the presence of depressive symptoms indicated by a total score of ≥8 (45–47).

### 2.5 Statistical analysis

Continuous variables are expressed as mean ± standard deviation (SD), while categorical variables are expressed as frequency (percentage). The demographic characteristics between cognitive impairment and non-cognitive impairment groups were compared using chi-square test for categorical variables and t-tests for continuous variables.

Multivariate logistic regression models were used to analyze the associations of sleep duration and quality with cognitive impairment. Sleep quality were included in the models with dichotomized variables (no and yes), continuous variables (the global PSQI scores), and the 6 aspects (in addition to sleep duration), respectively. Based on the published literature, confounders were sequentially added into the 4 models: model 1 included age and gender; model 2 included the variables in model 1 plus residence, education, occupation, marital status, living arrangements and economic situation; model 3 included the variables in model 2 plus drinking, smoking, BMI, and chronic disease; and model 4 included the variables in model 3 plus further adjustment for depressive symptoms. Model 4 was also run to rule out the case that the effect was mainly driven by significant depressive symptoms. Data were presented as unstandardized betas and 95% confidence intervals.

To assess the impact of gender and age, subgroup analyzes were also performed based on the following pre-specified characteristics: gender (male vs. female) and age (60–70 years vs. 71–80 years vs. 81–97 years). In addition, the dose–response curve is plotted through a restricted cubic spline which adopts sleep deprivation as a continuity variable. All the analyzes and drawing were performed using SPSS 20.0, R3.6.1 and GraphPad Prism Version 8.0, with *p* < 0.05 indicating statistically significant differences.

## 3 Results

### 3.1 Participant characteristics

The baseline characteristics of the participants are summarized in Table 1. For the 4,837 enrolled individuals, the mean age was 71.13 ± 5.50 years, and 51.09% were female. A total of 1,811 participants (aged 72.01 ± 5.93 years) were found to have cognitive impairment, with a prevalence of 37.44%. The participants at an older age (72.01 ± 5.93 years) and females (42.33%, *χ*^2^_gender_ = 51.582, *p* < 0.001) were more likely to have cognitive impairments. As shown in Table 1, there are statistically significant differences in cognitive impairment between age groups (*χ*^2^_age_ = 69.01, *p* < 0.001). More participants living in rural areas had cognitive impairment compared to those living in urban areas (39.15% vs. 28.92%). Significant differences were also found between different levels of education and different occupations (*χ*^2^_education_ = 25.602, *χ*^2^_occupation_ = 15.012, *p* < 0.001). Nearly half of the participants (48.14%, 181/376) who were single had cognitive impairment, and the proportion was significantly higher than that of the participants who were married (36.54%, 1630/4461; *χ*^2^_marriage_ = 19.920, *p* < 0.001). Significant differences were also found between participants with different economic situation (*χ*^2^ = 14.506, *p* < 0.001) and health behavioral factors (*χ*^2^_staple_ = 10.246, *p* < 0.006; *χ*^2^_drinking_ = 33.760, *p* < 0.001; *χ*^2^_smoking_ = 20.377, *p* < 0.001; *χ*^2^_BMI_ = 10.160, *p* < 0.017). However, no significant difference was found in the proportion of participants with cognitive impairment between groups with and without diabetes and between groups with and without COPD (*χ*^2^_diabetes_ = 0.953, *χ*^2^_COPD_ = 0.001, *p* > 0.05).

**Table 1:** Baseline characteristics of the participants with intact cognition and cognitive impairment.

Characteristics	Total Samples [*n* (%)]	Cognitive impairment			
		Yes [*n* (%)]	No [*n* (%)]	*χ*^2^/*t*	*p* value
No. of subjects					
Age (years, mean, SD)	71.13 ± 5.50	72.02 ± 5.93	70.59 ± 5.15	8.786	<0.001
Gender					
Male	2,366 (48.91)	765 (32.33)	1,601 (67.67)	51.582	<0.001
Female	2,471 (51.09)	1,046 (42.33)	1,425 (57.67)		
Age group (year)					
60–70	2,495 (51.58)	831 (33.31)	1,664 (66.69)	69.01	<0.001
71–80	2047 (42.32)	813 (39.72)	1,234 (60.28)		
81 ~ 97	295 (6.10)	167 (56.61)	128 (43.39)		
Residence					
Urban	809 (16.73)	234 (28.92)	575 (71.08)	30.079	<0.001
Rural	4,028 (83.27)	1,577 (39.15)	2,451 (60.85)		
Education					
Illiterate	2,628 (54.33)	1,064 (40.49)	1,564 (59.51)	25.602	<0.001
Primary school	1,296 (26.79)	457 (35.26)	839 (64.74)		
Junior school or above	913 (18.88)	290 (31.76)	623 (68.24)		
Occupation					
Employed knowledge worker	216 (4.47)	59 (27.31)	157 (72.69)	15.012	<0.001
Employed manual worker	2,179 (45.05)	789 (36.21)	1,390 (63.79)		
Others	2,442 (50.49)	963 (39.43)	1,479 (60.57)		
Marital status					
Married	4,461 (92.23)	1,630 (36.54)	2,831 (63.46)	19.920	<0.001
Single	376 (7.77)	181 (48.14)	195 (51.86)		
Living arrangements					
Living alone	205 (4.24)	89 (43.41)	116 (56.59)	3.262	0.077
Living with others	4,632 (95.76)	1722 (37.18)	2,910 (62.82)		
Economic situation^#^					
Affluent	90 (1.86)	24 (26.67)	66 (73.33)	14.506	<0.001
Ordinary	3,600 (74.43)	1,301 (36.14)	2,299 (63.86)		
Poor	991 (20.49)	413 (41.68)	578 (58.32)		
Health behavioral factors					
Staple food^#^					
Rice	118 (2.44)	40 (33.33)	80 (66.67)	10.246 0.006	
Farina	2,453 (50.71)	972 (39.62)	1,491 (60.36)		
Rice and farina	2,203 (45.54)	776 (35.22)	1,440 (64.89)		
Vegetable^#^					
Daily	4,438 (91.75)	1,674 (37.72)	2,764 (62.28)	1.362	0.250
No/occasionally	311 (6.43)	107 (34.41)	204 (65.59)		
Drinking					
Never	3,765 (77.86)	1,490 (39.58)	2,275 (60.42)	33.760	<0.001
Occasionally	482 (9.96)	151 (31.33)	331 (68.67)		
Every day	590 (12.20)	170 (28.81)	420 (71.19)		
Smoking^#^					
Non-smoker	3,287 (67.96)	1,301 (39.58)	1986 (60.42)	20.377	<0.001
Former smoker	969 (20.03)	317 (32.71)	652 (67.29)		
Current smoker	552 (11.41)	182 (32.97)	370 (67.03)		
BMI (kg/m^2^)^a^					
<18.5	583 (12.05)	246 (42.20)	337 (57.80)	10.160	0.017
18.5–23.99	1902 (39.32)	725 (38.12)	1,177 (61.88)		
24.0–27.99	1,626 (33.62)	569 (34.99)	1,057 (65.01)		
>28.0	726 (15.01)	271 (37.33)	455 (62.67)		
Chronic diseases					
Diabetes^#^					
No	4,229 (87.43)	1,574 (37.22)	2,655 (62.78)	0.953	0.342
Yes	593 (12.26)	233 (39.29)	360 (60.71)		
Hypertension					
No	1920 (39.69)	665 (34.64)	1,255 (65.36)	10.696	0.001
Yes	2,917 (60.31)	1,146 (39.29)	1771 (60.71)		
CHD^# b^					
No	3,925 (81.15)	1,439 (36.66)	2,486 (63.34)	5.997	0.016
Yes	899 (18.59)	369 (41.05)	530 (58.95)		
Stroke^#^					
No	4,119 (85.16)	1,521 (36.93)	2,598 (63.07)	3.676	0.058
Yes	705 (14.58)	287 (40.71)	418 (59.29)		
COPD^# c^					
No	4,382 (90.59)	1,642 (37.47)	2,740 (62.53)	0.001	1.000
Yes	440 (9.10)	165 (37.50)	275 (62.50)		
Sleep quality					
Good (≤7)	3,082 (63.72)	1,063 (34.49)	2019 (65.51)	31.560	<0.001
Poor (>7)	1755 (36.28)	748 (42.62)	1,007 (57.38)		
Duration of sleep(h)					
<6	953 (19.7)	366 (38.4)	578 (61.6)	20.752	<0.001
6 ~ <7	993 (20.5)	387 (39.0)	606 (61.0)		
7 ~ <8 8 ~ <9	1,058 (21.9) 1,038 (21.5)	336 (31.8) 395 (38.1)	722 (68.2) 643 (61.9)		
≥9	795 (16.4)	327 (41.1)	468 (58.9)		
Depressive symptoms^#^					
Yes	549 (11.35)	273 (49.73)	276 (50.27)	39.951	<0.001
No	4,207 (86.98)	1,508 (35.85)	2,699 (64.15)		

^a^Body mass index is calculated as weight in kilograms divided by height in meters squared. ^b^CHD: Coronary heart disease; ^c^COPD: chronic obstructive pulmonary disease. #4,681 participants (96.8%) had data of economic situation, 4,774 (98.7%) had data of staple food, 4,749(98.2%) had data of vegetable eating, 4,808 (99.4%) had data of smoking, 4,822 (99.7%) had data of diabetes, 4,824 (99.7%) had data of coronary heart disease, 4,824 (99.7%) had data of stroke, 4,822 (99.7%) had data of chronic obstructive pulmonary disease, and 4,756 (98.3%) had data of depressive symptoms.

Among the 1,811 participants with cognitive impairment, 173 (38.27%) had very short sleep duration (<6 h.), 580 (38.82%) had short sleep duration (6–6.9 h.), 327 (41.13%) had long sleep duration (>9 h), and 731 (34.88%) were in the reference group (7–8.9 h.). The prevalence of poor sleep quality was 33.28% in participants with intact cognition and 41.30% in those with cognitive impairment. The proportion of participants with cognitive impairment is higher among those with poor sleep quality (42.62%, 748/1755), compared with those with good sleep quality (34.49%, 1063/3082, *χ*^2^_sleep quality_ = 31.560, *p* < 0.001). The proportion of participants with cognitive impairment is higher in those with symptoms of depression (49.73%, 273/549), as compared with those without symptoms of depression (35.85%, 1508/4207; *χ*^2^_depression_ = 39.951, *p* < 0.001).

### 3.2 Cross-sectional analysis of sleep duration and sleep quality with cognitive impairment

Model 4 revealed that, compared to 7–7.9 h., the multivariable adjusted odds ratio (OR) for cognitive impairment was 1.28 (95%CI, 1.053–1.557) for<6 h., 1.425 (95%CI, 1.175–1.728) for 6–6.9 h., 1.294 (95%CI, 1.068–1.566) for 8–8.9 h., and 1.360 (95%CI, 1.109–1.668) for ≥9 h. of sleep duration (Table 2). With regard to sleep quality, compared to participants with good sleep quality, the full adjusted OR and 95%CI of cognitive impairment for those with poor sleep quality was 1.263 and 1.108–1.440. For the seven subscales of PSQI, no significant association was found (in addition to daytime dysfunction; OR 1.128, 95%CI 1.055–1.207) when specific factors (depressive symptoms) were included as covariates.

**Table 2:** Logistic regression analysis of the correlations between sleep duration as well as sleep quality and cognitive impairment.

Sleep status	Model 1		Model 2		Model 3		Model 4	
	OR (95%CI)	*P* value	OR (95%CI)	*P* value	OR (95%CI)	*P* value	OR (95%CI)	*P* value
Sleep duration (h)								
<6	1.327 (1.101–1.599)	0.030	1.292 (1.066–1.566)	0.009	1.301 (1.073–1.578)	0.008	1.280 (1.053–1.557)	0.013
6 ~ <7	1.396 (1.161–1.679)	<0.001	1.423 (1.177–1.720)	<0.001	1.414 (1.168–1.710)	<0.001	1.425 (1.175–1.728)	<0.001
7 ~ <8	ref		Ref		Ref		Ref	
8 ~ <9	1.317 (1.097–1.582)	0.030	1.257 (1.042–1.518)	0.017	1.272 (1.053–1.537)	0.013	1.294 (1.068–1,566)	0.008
≥9	1.433 (1.179–1.741)	<0.001	1.335 (1.092–1.632)	0.005	1.337 (1.092–1.636)	0.005	1.360 (1.109–1.668)	0.003
Dichotomized (Overall sleep quality)								
Good (≤7)	ref		ref		ref		ref	
Poor (>7)	1.346 (1.190–1.523)	<0.001	1.320 (1.162–1.498)	<0.001	1.307 (1.149–1.485)	<0.001	1.263 (1.108–1.440)	0.001
Continuous (PSQI score)								
The global PSQI	1.034 (1.017–1.051)	<0.001	1.032 (1.014–1.049)	<0.001	1.030 (1.012–1.047)	0.001	1.024 (1.007–1.042)	0.007
Subjective sleep quality	1.133 (1.037–1.238)	0.006	1.116 (1.018–1.223)	0.019	1.103 (1.006–1.210)	0.038	1.078 (0.981–1.184)	0.121
Sleep latency	1.078 (1.025–1.134)	0.004	1.070 (1.016–1.128)	0.011	1.067 (1.012–1.124)	0.016	1.052 (0.998–1.110)	0.061
Sleep efficiency	1.067 (1.011–1.125)	0.017	1.066 (1.009–1.127)	0.022	1.064 (1.006–1.124)	0.030	1.053 (0.995–1.114)	0.075
Sleep disturbance	1.077 (0.969–1.198)	0.168	1.048 (0.939–1.169)	0.401	1.039 (0.930–1.161)	0.499	1.011 (0.903–1.132)	0.848
Sleep medications	1.100 (0.973–1.244)	0.128	1.135 (1.001–1.288)	0.048	1.122 (0.988–1.275)	0.075	1.112 (0.977–1.265)	0.108
Daytime dysfunction	1.180 (1.108–1.256)	<0.001	1.148 (1.075–1.226)	<0.001	1.141 (1.068–1.219)	<0.001	1.128 (1.055–1.207)	0.001

PSQI, Pittsburgh Sleep Quality Index; Model 1 included age and gender; Model 2 included model 1 plus residence, education, occupation, marital status, living arrangements, and economic situation; Model 3 included model 2 plus drinking, smoking, BMI, and chronic disease; Model 4 included model 3 plus Depressive symptoms.

### 3.3 Association of sleep duration and sleep quality with cognitive impairment stratified by gender and age

**Figure 2:**
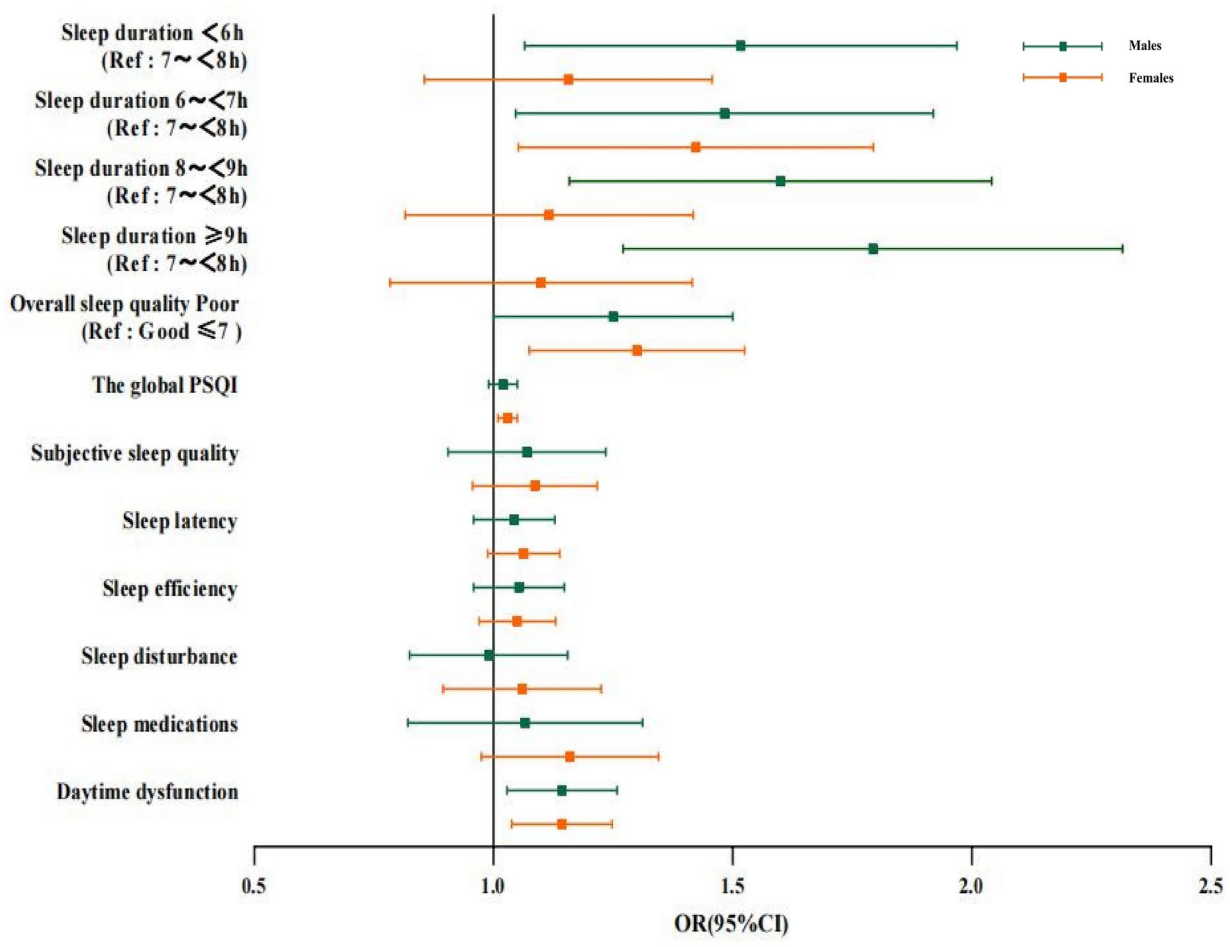
Logistic regression analysis of the correlation between sleep duration, sleep quality and cognitive impairment in males and females. Data of different genders were analyzed based on model 4. OR, Odds ratio; CI, confidence interval; PSQI: Pittsburgh Sleep Quality Index; Ref: reference.

The relationship between sleep duration and cognitive impairment was different between the two gender groups (Figure 2). Interestingly, excessively short or long sleep duration was associated with cognitive impairment in males (P<0.05). Whereas in females, compared to 7–7.9 h. of sleep duration, the full adjusted OR and 95%CI of cognitive impairment for the sleep duration of 6–6.9 h. were 1.393 and 1.074–1.808, and no significant statistical difference was found between individuals reporting a sleep duration of <6 h., 8–8.9 h., and ≥ 9 h. among females.

**Figure 3:**
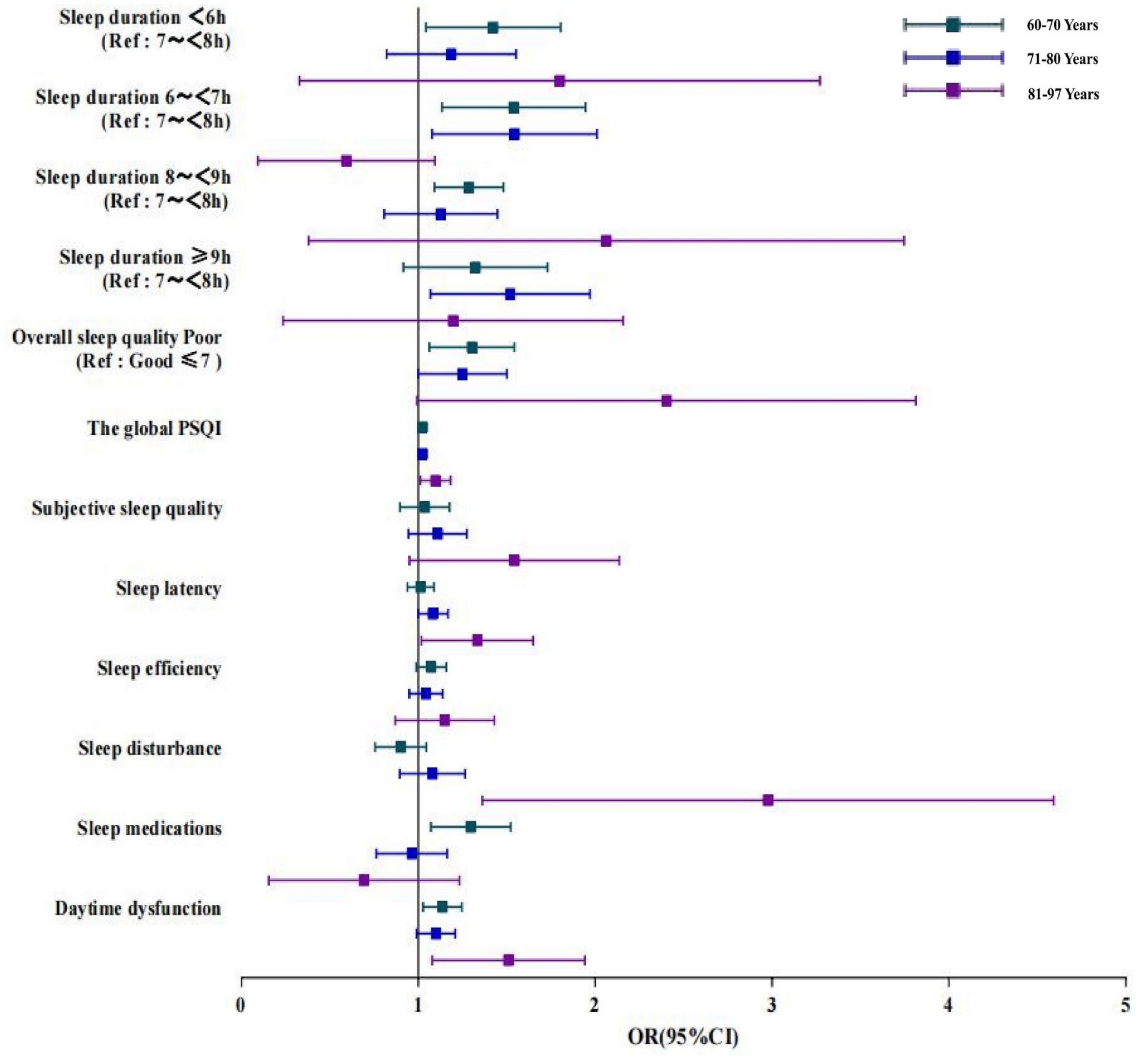
Logistic regression analysis of the correlation between sleep duration, sleep quality and cognitive impairment in older adult people of different ages. Data of different ages were analyzed based on Model 4. OR, Odds ratio; CI, confidence interval; PSQI: Pittsburgh Sleep Quality Index; Ref: reference.

The association of poor sleep quality (including daytime dysfunction) with poor cognitive function was found in subgroup analyzes of gender. It was found that poor sleep quality was significantly correlated with cognitive impairment in all age groups, with the association being stronger in the group at a higher age (OR: 2.128, 95%CI: 1.152–3.934; Figure 3). Furthermore, with regard to the subscales measuring sleep quality, the groups aged 81–97 years with daytime dysfunction had higher odds cognitive impairment than other age groups. We also found that the use of sleep medication was positively associated with cognitive impairment in the group aged 60–70 years, with an OR (95%CI) of 1.284 (1.079, 1.529), while sleep latency was associated with an increased risk of cognitive impairment for the group aged 81–97 years, with an OR (95%CI) of 1.305 (1.027, 1.659). The association of sleep duration with cognitive impairment was more obvious in the 60-to-70-year-old group (<6 h., OR 1.388, 95%CI: 1.059–1.820; 6–6.9 h., OR 1.505, 95%CI: 1.154–1.961; 8–8.9 h., OR 1.510, 95%CI: 1.145–1.990, respectively) and the 71-to-80-year-old group (6–6.9 h., OR 1.498, 95%CI, 1.104–2.034; ≥9 h., OR 1.475, 95%CI: 1.092–1.992, respectively); however, such an association was not significant in groups with older age.

**Figure 4:**
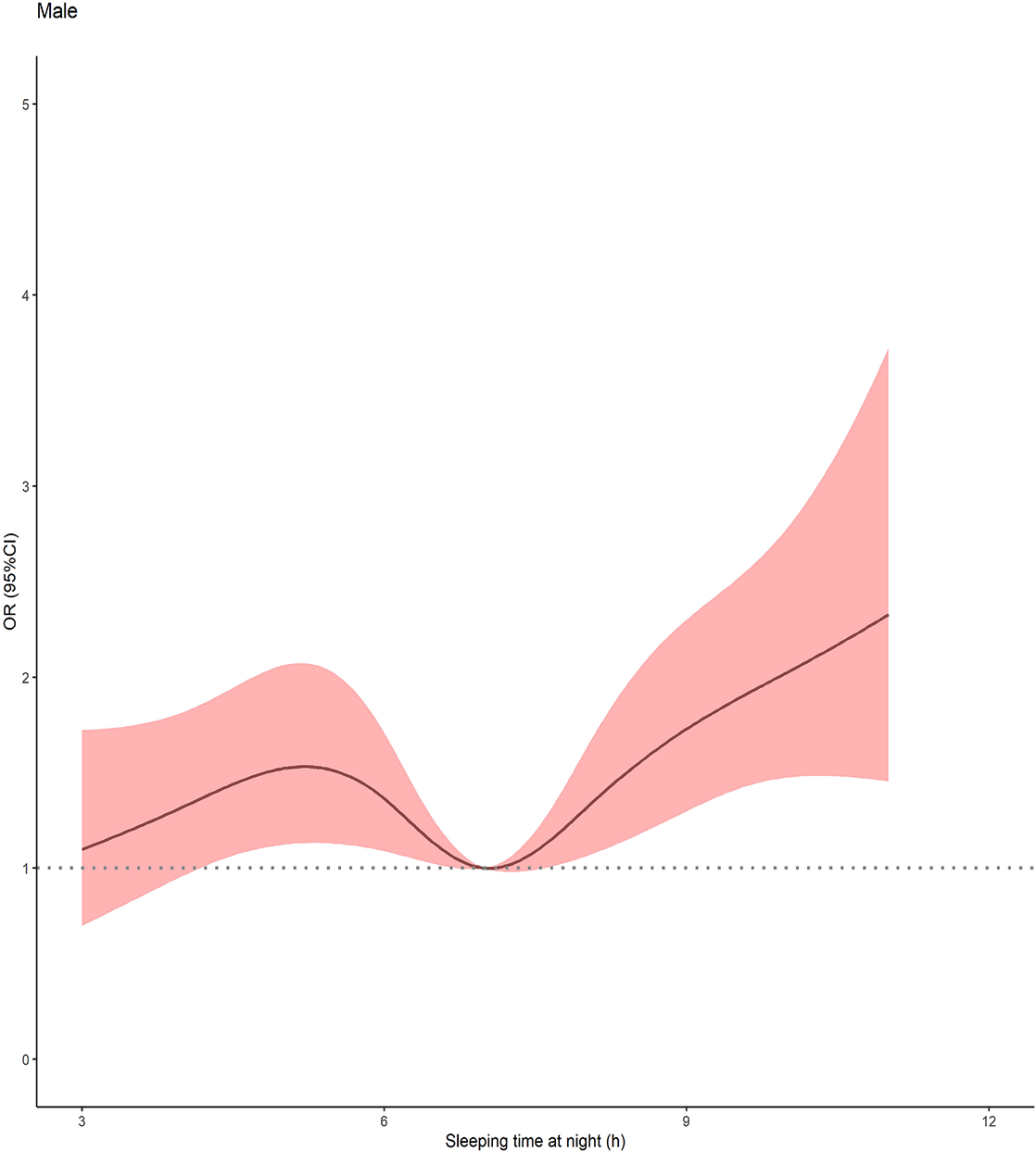
Multivariable-adjusted spline curve for association of sleep duration with cognitive impairment (*n* = 4,837). Solid line represented Odds ratio of cognitive impairment, adjusting for age, residence, education, occupation, marital status, living arrangements, economic situation, drinking, smoking, BMI, chronic disease and depressive symptoms. The shaded area represented the 95%CI. The histogram represented the distribution of study participants. CI, confidence interval.

Furthermore, the restricted cubic spline fitted to sleep deprivation and cognitive decline was used to show the dose–response relationship between the sleep duration and cognitive decline. As shown in Figure 4, a V-shaped association between sleep duration and cognitive impairment was found in a dose–response curve.

## 4 Discussion

### 4.1 Main findings

In this large community-based study of people aged 60 years and older in China, we observed the associations between sleep duration, sleep quality and cognitive performance, and explored whether gender and age affected these associations. Cross-sectional analyzes revealed that both inadequate or excessive sleep and poor sleep quality were significantly associated with increased risk of cognitive impairment. Specifically, subgroup analysis revealed a V-shaped association between self-reported sleep duration and cognitive impairment among males. Poor sleep quality, including daytime dysfunction, was also associated with cognitive impairment, with these effects being the strongest in females and the group aged 81–97 years.

### 4.2 Comparison with previous work

In the present study, the prevalence of cognitive impairment among older adults aged 60 years and above in Fuyang, Anhui Province was 37.44%, which was lower than those reported by Lv et al. (38.5%) (48) and Liu et al. (50%) (49) and higher than that reported by Zhang et al. (24.8%) (50). The great disparity in the prevalence of cognitive impairment might be partly attributed to differences in research methods as well as the level of education and age composition of the study subjects.

The strength of the association between sleep duration and cognitive impairment varied across different groups. Numerous prior studies have shown that excessively short (9, 17, 22, 51) or long sleep duration can increase the risk of cognitive impairment (52–54), which is in line with our findings. Notably, a cohort study using the United Kingdom Biobank suggests that there is a U-shaped association between sleep duration and the risk of dementia, with excessively long or short sleep duration associated with a higher risk of brain atrophy and a sleep duration of 7 h./day associated with a lower risk of dementia (55); this also supports our findings. Previous studies suggested that insufficient or excessive sleep at night could increase the risk of cognitive impairment (56). A meta-analysis involving cross-sectional and prospective studies on self-reported sleep duration and cognitive performance revealed that both insufficient and excessive sleep in older age (>55 years) was associated with cognitive impairment (57). A longitudinal cohort study on people aged ≥50 based in Mexico found a significant association between long sleep duration and an oval-shaped decline of cognitive function (11). Furthermore, a cohort study found that excessive sleep (>8 h./night) was associated with an increase in the risk of all-cause dementia by 69% (58). Other studies also found a similar association. A study based on the database of Chinese Longitudinal Healthy Longevity Survey suggested that there was a U-shaped association between sleep duration and cognitive disorder, i.e., both excessively short and long sleep was associated with cognitive disorder (59). Another long-term study, which involved 7,342 middle-aged and older adult people in China, also found a U-shaped association of the total sleep duration and nocturnal sleep duration with cognitive function, suggesting that excessively short or long sleep could increase the risk of cognitive disorder. Studies also found that people with a total sleep duration of 7–8 h per day and a nocturnal sleep duration of 6–7 h are at the lowest risk of cognitive disorders (49). A national cohort study found that increased sleep duration among older adult people was independently associated with cognitive decline, and could be affected by gender and level of education (60). Therefore, subjective measurement of long sleep duration in elder people might be an important indicator for the risk of dementia. Poor sleep quality, including greater daily dysfunction and the use of higher dose of sleep medication (s) due to poor sleep quality, has been reported to be associated with a higher risk of cognitive impairment (28, 61). However, a cohort study found no association between the PSQI global score and cognitive function (47). Such disparity might have resulted from the difference between the self-reported overall sleep quality and the global PSQI score, which measure sleep quality qualitatively and quantitatively, respectively. In the present study, poor sleep quality, including poor overall sleep quality (OR: 1.263, 95%CI: 1.108–1.440) and higher global PSQI score (OR: 1.024, 95%CI: 1.007–1.042), was associated with an increased risk of cognitive impairment, compared with good sleep quality. Moreover, subscale scores in the PSQI showed no significant association with cognitive impairment when a specific factor, depressive symptoms, was included as a covariate. For older people with poor sleep quality, the decline of cognitive function might also be associated with daytime dysfunction, in addition to the duration of sleep. Our findings are consistent with a study involving 695 senior participants with type 1 diabetes, which showed an association between poor sleep quality and lower global cognition as well as a strong association of global cognitive function with the use of sleep medications and daytime dysfunction (62). Studies have also reported that daytime dysfunction (63) and the use of sleep medications (64) were associated with an increased risk of cognitive impairment. In contrast to the above studies, the result from a nationally-representative cohort of older adults from the US found no evidence supporting that self-reported sleep duration or sleep quality was a significant contributor to cognitive function (65).

### 4.3 Possible explanations

At present, the biological mechanism underlying the association between sleep duration, sleep quality and cognitive impairment remains unclear. Previous studies showed that the level of melatonin and circadian rhythm dysregulation might play an important role (53, 66), and a recent study found that melatonin and its two metabolites might help maintain memory and protect mice and humans from cognitive impairment (67). An experimental study found that the accumulation of amyloid-β in mice and humans could result in sleep disorders (68), and studies also indicated that subjective indicators such as poor sleep quality were correlated with an increased cortical amyloid-β burden, and that the level of amyloid-β in the cerebrospinal fluid was correlated with the level of phosphorylated tau (69, 70). Recent data showed that insufficient or disrupted sleep could result in an increase of inflammatory cells (71), which might affect the association between sleep and the risk for dementia (72). Additionally, relevant studies speculated that poor sleep quality under different medical conditions might be related to disturbed brain insulin-like growth factor-I (IGF-I) input into orexin neurons (73). Another study found that an increased orexin level of in the cerebrospinal fluid was related to poor sleep condition, which appeared to be associated with cognitive impairment in patients with AD (74). A small sample study explored the association of sleep quality and duration with the brain microstructure of older adult people, which revealed that poor sleep quality and extreme sleep duration were associated with cerebral atrophy and dementia; this study also revealed gender differences, i.e., poor sleep quality among males were more strongly associated with abnormal microstructure, while excessively short or long sleep among females was associated with abnormal brain microstructure (75). Some studies also pointed out that abnormal sleep-related indicators (e.g., sleep duration and sleep quality) could increase the risk of hypertension and coronary heart diseases (76, 77), which might be another cause of increased risk for cognitive impairment in older adult people (78–80). However, in the present study, the correlation between sleep duration, sleep quality and cognitive function did not change significantly after further adjustment for chronic diseases, indicating that chronic medical conditions might not be key factors affecting cognitive function.

### 4.4 Stratified analysis by gender and age

The strength of association between sleep duration and cognitive impairment varied across different gender and age groups. Compared with females and the 81-to-97-year-old group, the correlation between sleep duration and cognitive impairment was more pronounced in males and the group aged 60–80 years. In the subgroup analysis, the strength of correlation between sleep duration and cognitive function is different between males and females, with the correlation in males showing a significant V-shaped association. Compared to the reference group (i.e., those with a sleep duration of 7–7.9 h.), the association between sleep duration and cognitive impairment was more obvious in the 60-to-70-year-old group with a sleep duration of <6 h., 6–6.9 h. and 8–8.9 h., as well as in the 71-to-80-year-old group with a sleep duration of 6–6.9 h. and ≥ 9 h. According to a literature review, declined gonadal function in elder females and males is associated with sleep disorders, cognitive impairment and dementia, indicating that gender difference and gonadal steroid hormones might play a role in the regulation of the above conditions (81), but the specific neuroendocrine mechanism underlying their vulnerability to the harmful effects is yet to be elucidated. Another study found that insufficient sleep in men and excessive sleep in women could increase the risk of MCI (36). A retrospective longitudinal analysis revealed that short sleep duration and sleep variability were associated with cognitive impairment in the sample of older adult people in the community; it was also found that the female gender, a lower level of education, the presence of at least one APOE4 allele and a higher level of depression were associated with increased risk of cognitive impairment (82). However, the mechanism related to the gender difference regarding the association between sleep problems and cognitive function is still unclear, thus, further studies are needed to investigate this complex interaction. A cross-sectional study on Chinese elderlies dwelling in rural areas found that the association of dementia with inadequate or excessive sleep and poor sleep efficiency was present only in older adult people aged <75 years rather than in those aged ≥75 years; this might be related to the survivorship bias, i.e., those at an older age and with comorbidities of sleep disorders and dementia were at a higher risk of death (83). Another reason can be that the cognitive function of individuals at an advanced age might be affected by other factors, such as physical conditions, mood disorders, and social support, resulting in a weakened contribution of sleep duration to the influence on cognitive dysfunction. However, the features of sleep reported by elder people at different age groups might be different, which might affect the strength of association between sleep problems and cognitive impairment. Collectively, the strength of the association between sleep duration and cognitive impairment varies across different gender and age groups, and further studies are needed to explore the underlying mechanism. In addition, significant correlation between poor sleep quality (including daytime dysfunction) and cognitive impairment was found in different gender groups, and significant association between daytime dysfunction and cognitive impairment was also found in subgroup analyzes, with the 81-97-year-old group with daytime dysfunction having higher odds than other age groups. Unexpectedly, a higher global PSQI score (including sleep latency) was also found associated with an increased risk of cognitive impairment among individuals aged 81–97 years. We also found that the use of sleep medications was positively correlated with cognitive impairment in the group aged 60–70 years. A cohort study in the United Kingdom suggested that inadequate sleep had the greatest contribution to subsequent dementia among individuals in their 50s and 60s, while it only led to a higher risk of dementia among individuals in their 70s who were free from any mental disorders (51); this study indicated that, for people at an advanced age, the influence of sleep on cognitive function might not be as significant as other physical conditions.

### 4.5 Implications for public health

Given the critical role of cognitive health in the aging population, our findings may have important implications for public health. This study reveals a positive association between insufficient or excessive sleep and cognitive impairment, particularly among men. This implies that targeted interventions and policies to regulate sleep duration in older adults are necessary to protect the cognitive health of older adults and specific populations. Furthermore, our study emphasizes the intricate relationship between sleep quality and cognitive health, including daytime dysfunction. The significant correlation between poor sleep quality and cognitive impairment, particularly in females and the 81–97 age group, underscores the need for interventions targeting sleep quality to prevent cognitive decline, which provides another effective way. Overall, our findings have direct implications for public health strategies aimed at promoting comprehensive well-being in older populations.

### 4.6 Strengths and limitations

With the use of a large community-based sample of the older adult population and adjustment of a variety of important confounders, the results of the present study are relatively robust. However, as this study is cross-sectional, the results cannot demonstrate causal relationships, which need verification in further prospective studies. As the information on the duration of nighttime sleep was obtained from subjective reports, objective measurement of sleep-related indicators is also needed in future studies as sleep characteristics obtained from objective sleep monitoring, along with multidimensional cognitive assessment, can help to yield more convincing results. In addition, although some factors were controlled for in the multivariate model, more stringent controlling for the residual confounders is also needed in future studies. Some factors likely to affect an individual’s sleep pattern and cognitive function, such as obstructive sleep apnea, were not involved in this study; thus, further studies are warranted to explore such factors in a more comprehensive way.

In the future, longitudinal studies are needed to explore the causal relationship between sleep duration and quality and cognitive performance. Future works may also explore whether improving sleep quality and/or duration can reduce the risk of cognitive impairment. In further studies, we will continue collecting data on sleep and cognitive function using scales and experiments. Other moderators, such as anxiety (84, 85), health-related factors (86, 87), serum biomarkers (72, 88, 89) and environmental exposures (90) are also of potential value for future studies.

## 5 Conclusion

In summary, the present study showed that abnormal sleep duration was an independent factor associated with cognitive impairment, especially with a significant V-shaped association in males. Cognitive impairment is associated with poor sleep quality and daytime dysfunction, with this effect being the strongest in females and individuals aged 81–97 years. Our results suggest that appropriate sleep duration and high-quality sleep quality may be important for maintaining cognitive function in the older adults and specific populations. Further cohort studies are needed to clarify these associations, and to develop a better understanding of the mechanisms.

## Data availability statement

The original contributions presented in the study are included in the article/supplementary material, further inquiries can be directed to the corresponding authors.

## Ethics statement

The study was conducted in accordance with the Declaration of Helsinki and was approved by the Ethical Review Committee of Anhui Medical University. The studies were conducted in accordance with the local legislation and institutional requirements. The participants provided their written informed consent to participate in this study. Written informed consent was obtained from the individual(s) for the publication of any potentially identifiable images or data included in this article.

## Author contributions

XL and PX contributed to the drafting of manuscript, formal analysis and data curation. RW, BC, and LS contributed to the investigation and data curation and validation. LY and GC conceived, designed, and supervised all aspects of the study and revised the manuscript. All authors contributed to the article and approved the submitted version.
